# Supplementary material for: Understanding the CH4 Conversion over Metal Dimers from First Principles
Source: Nanomaterials (Basel). 2022 Apr 29;12(9):1518. doi: 10.3390/nano12091518 (PMC9100024; doi:10.3390/nano12091518)
Supplement: Supplementary file 1 [file nanomaterials-12-01518-s001.zip › nanomaterials-1676240-supplementary.pdf]

nanomaterials

MDPI

Supplemental Information

Understanding the CH_4 Conversion over Metal Dimers from First Principles

Haihong Meng [1], Bing Han [1], Fengyu Li [1,*], Jingxiang Zhao [2,*] and Zhongfang Chen [3,*]

[1] School of Physical Science and Technology, Inner Mongolia University, Hohhot 010021, China; dundun_0521@163.com (H.M.); binghan1214@163.com (B.H.)

[2] College of Chemistry and Chemical Engineering, Key Laboratory of Photonic and Electronic Bandgap Materials, Ministry of Education, Harbin Normal University, Harbin 150025, China

[3] Department of Chemistry, The Institute for Functional Nanomaterials, University of Puerto Rico, Rio Piedras Campus, San Juan, PR 00931, USA

* Correspondence: fengyuli@imu.edu.cn (F.L.); xjz_hmily@163.com (J.Z.); zhongfangchen@gmail.com (Z.C.)

Table S1. The lattice parameters (*a*/*b*, in Å) of M_2-Pc catalysts, their corresponding bond lengths (the bond lengths of metal to metal, metal to nitrogen, d_{M-M}, d_{M-N}, d_{M-Nc}, where Nc represents the N atom adjacent to the C atom, in Å), and the binding energy (E_b, in eV) of metal dimer anchoring at Pc monolayer, as well as the cohesive energy (E_{bulk}, in eV) of metal in bulk.

M	*a*/Å	*b*/Å	d_{M-M}/Å	d_{M-N}/Å	d_{M-Nc}/Å	E_b/eV	E_{bulk}/eV
Sc	14.19	14.26	2.95	2.08	2.05	-11.50	-6.20
Ti	14.16	14.22	2.66	2.02	1.94	-12.15	-7.76
V	14.13	14.18	2.56	2.00	1.90	-11.29	-9.76
Cr	14.23	14.25	2.52	1.94	1.86	-9.66	-9.49
Mn	14.18	14.19	2.38	1.95	1.82	-9.66	-8.94
Fe	14.13	14.14	2.33	1.93	1.78	-10.06	-8.30
Co	14.10	14.12	2.37	1.91	1.77	-10.52	-7.11
Ni	14.17	14.18	2.73	1.83	1.83	-9.92	-5.57
Cu	14.23	14.25	2.82	1.86	1.89	-7.26	-4.39
Zn	14.21	14.27	2.84	1.93	1.98	-5.04	-1.26
Y	14.18	14.25	3.25	2.22	2.22	-11.05	-6.32
Zr	14.22	14.27	2.95	2.12	2.08	-12.49	-8.48
Nb	14.17	14.23	2.57	2.11	2.00	-12.11	-10.22
Mo	14.16	14.22	2.50	2.06	1.96	-10.21	-10.95
Ru	14.26	14.28	2.43	2.00	1.91	-11.42	-9.20
Rh	14.27	14.28	2.49	1.98	1.90	-10.52	-7.26
Pd	14.23	14.30	2.84	1.94	1.98	-7.60	-5.16
Ag	14.25	14.31	2.79	1.99	2.06	-4.13	-3.37
Hf	14.21	14.27	2.94	2.08	2.05	-13.17	-9.96

Ta	14.19	14.25	2.72	2.07	2.01	-13.47	-11.86
W	14.18	14.22	2.52	2.05	1.97	-12.63	-13.01
Re	14.26	14.28	2.40	2.01	1.94	-12.06	-12.00
Os	14.24	14.25	2.42	1.98	1.92	-12.68	-11.24
Ir	14.27	14.28	2.51	1.98	1.92	-12.06	-8.85
Pt	14.25	14.32	2.89	1.93	1.97	-9.95	-6.04
Au	14.26	14.32	2.86	1.97	2.2	-5.84	-4.46
Al	14.21	14.23	2.71	1.86	1.85	-10.58	-5.52
Ga	14.29	14.31	2.78	1.90	1.90	-7.54	-3.45
Sn	14.18	14.25	3.27	2.29	2.23	-6.71	-4.38
Bi	14.18	14.26	3.33	2.25	2.24	-5.95	-4.68

Table S2a. The reaction energy for H_2O_2 dissociation into $*O + H_2O$ on the M_2-Pc (M = Sc, Zr, Nb, W), bond lengths of two metal atoms with oxygen (d_{M1-O}/d_{M2-O}, in Å) (M1 = M2 =M), as well as the H_2O binding energies ($E_{ads\,(H2O)}$, in eV).

	**Δ*E*/eV**	***d*$_{M1-O}$/Å**	***d*$_{M2-O}$/Å**	***E*$_{ads(H2O)}$/eV**
Sc	-27.12	1.98	1.97	-0.31
Zr	-26.83	2.05	2.05	-0.21
Nb	-25.91	2.00	2.00	-0.27
W	-23.52	2.06	1.96	-0.13

Table S2b. The reaction energy for H_2O_2 dissociation into $*OH + *OH$ on the M_2-Pc (M = Sc, Ti, V, Y, Zr, Hf, Ta, W), as well as the bond lengths (d_{M1-O}/d_{M2-O}, d_{O1-O2}, d_{O1-H}/d_{O2-H}, Å) (M1 = M2 = M), O1 and O2 represent the two O atoms bonded to M1 and M2, respectively.

	**Δ*E*/eV**	***d*$_{M1-O}$/Å**	***d*$_{M2-O}$/Å**	***d*$_{O1-O2}$/Å**	***d*$_{O1-H}$/Å**	***d*$_{O2-H}$/Å**
Sc	-5.19	1.93	1.92	2.99	0.97	0.97
Ti	-7.51	1.83	1.83	2.93	0.97	0.97
V	-6.70	1.79	1.79	2.98	1.03	0.98
Y	-5.03	2.08	2.07	3.10	0.97	0.97
Zr	-8.16	2.00	1.99	3.18	0.97	0.97
Hf	-8.71	1.95	1.96	2.97	0.97	0.97
Ta	-9.04	1.91	1.91	3.02	0.97	0.97
W	-7.41	1.95	1.88	2.76	1.00	0.98

Table S3. The total magnetic moments and magnetic moment (in μ_B) on the two metal atoms of the M_2-Pc (M = Sc, Ti, V, Y, Zr, Nb, Hf, Ta, W) (structures were shown in Figure S2), and the Bader charge (*q*, in |e|) of the two atoms.

	**total**	**M1**	**M2**	***q*$_{M1}$**	***q*$_{M2}$**
Nb	0.93	0.37	0.37	1.72	1.71

Sc	0.00	0.00	0.00	+1.86	+1.86
Ta	−0.02	0.45	−0.50	+1.79	+1.79
Ti	0.77	0.44	0.44	+1.78	+1.78
V	3.16	1.60	1.60	+1.57	+1.57
Y	0.00	0.00	0.00	+1.98	+1.98
Zr	0.39	0.22	0.22	+1.98	+1.98
Hf	0.39	0.20	0.20	+1.98	+1.98
W	0.00	0.69	−0.69	+1.60	+1.60

Table S4. The calculated zero-point energy, entropy, and free energy change of the reaction of the abstraction of a hydrogen from one of the *OH groups to the formation of water and oxo species ($*OH + *OH \rightarrow *O + H_2O$) on M_2-Pc (M = Ti, V, Y, Hf, Ta).

	**2*OH**		***O + H_2O**		**Δ*G*/eV**
	**ZPE**	**T*ΔS**	**ZPE**	**T*ΔS**	
Ti	0.65	0.21	0.67	0.25	0.91
V	0.69	0.21	0.74	0.16	0.74
Y	0.68	0.23	0.74	0.17	1.83
Hf	0.72	0.12	0.76	0.14	1.72
Ta	0.63	0.20	0.69	0.11	1.63

Table S5. The calculated zero-point energy, entropy, and free energy change of the self-reaction of H_2O_2 ($2*OH + H_2O_2 \rightarrow O_2 + 2H_2O$) on M_2-Pc (M = Sc, Ti, V, Y, Zr, Hf).

	**2*OH + H_2O_2**		**O_2 + 2H_2O**		**Δ*G*/eV**
	**ZPE**	**T*ΔS**	**ZPE**	**T*ΔS**	
Sc	1.43	0.40	1.39	0.45	0.54
Ti	1.45	0.42	1.41	0.45	2.61
V	1.46	0.39	1.40	0.33	2.69
Y	1.40	0.40	1.37	0.40	0.65
Zr	1.42	0.35	1.35	0.44	3.08
Hf	1.44	0.45	1.44	0.38	−0.18

Table S6. The partial charges of O (q_O) and M (q_{M1} and q_{M2}) for M1-O-M2 (in ∣e∣) (M1 = M2 = Sc, Zr, Nb, W). Values were obtained from Bader charge analysis.

	**q_{M1}**	**q_{M2}**	**q_O**
Sc1-O-Sc2	+1.89	+1.89	−1.14
Zr1-O-Zr2	+2.30	+2.30	−1.17
Nb1-O-Nb2	+2.10	+2.08	−1.07
W1-O-W2	+1.99	+2.03	−0.94

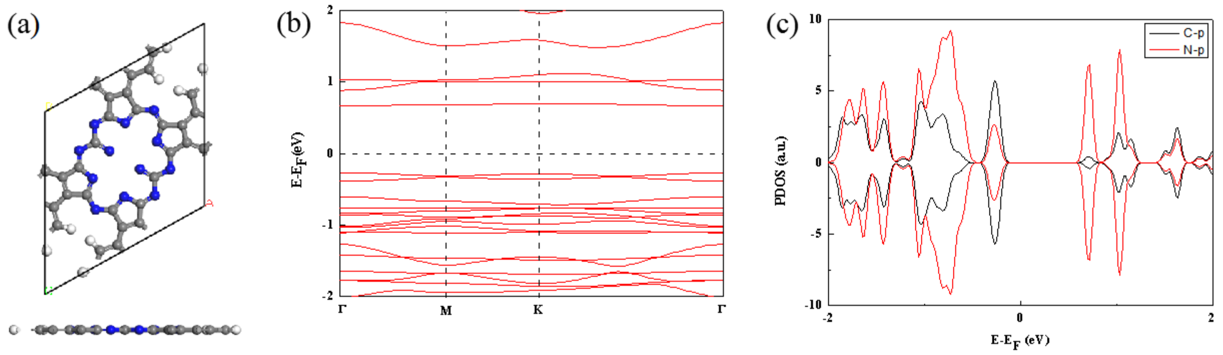

Figure S1. Top and side view of the structure of Pc in a 2×2×1 supercell (**a**), the band structure (**b**) and projected density of state (PDOS) (**c**). The Fermi lever is set to zero.

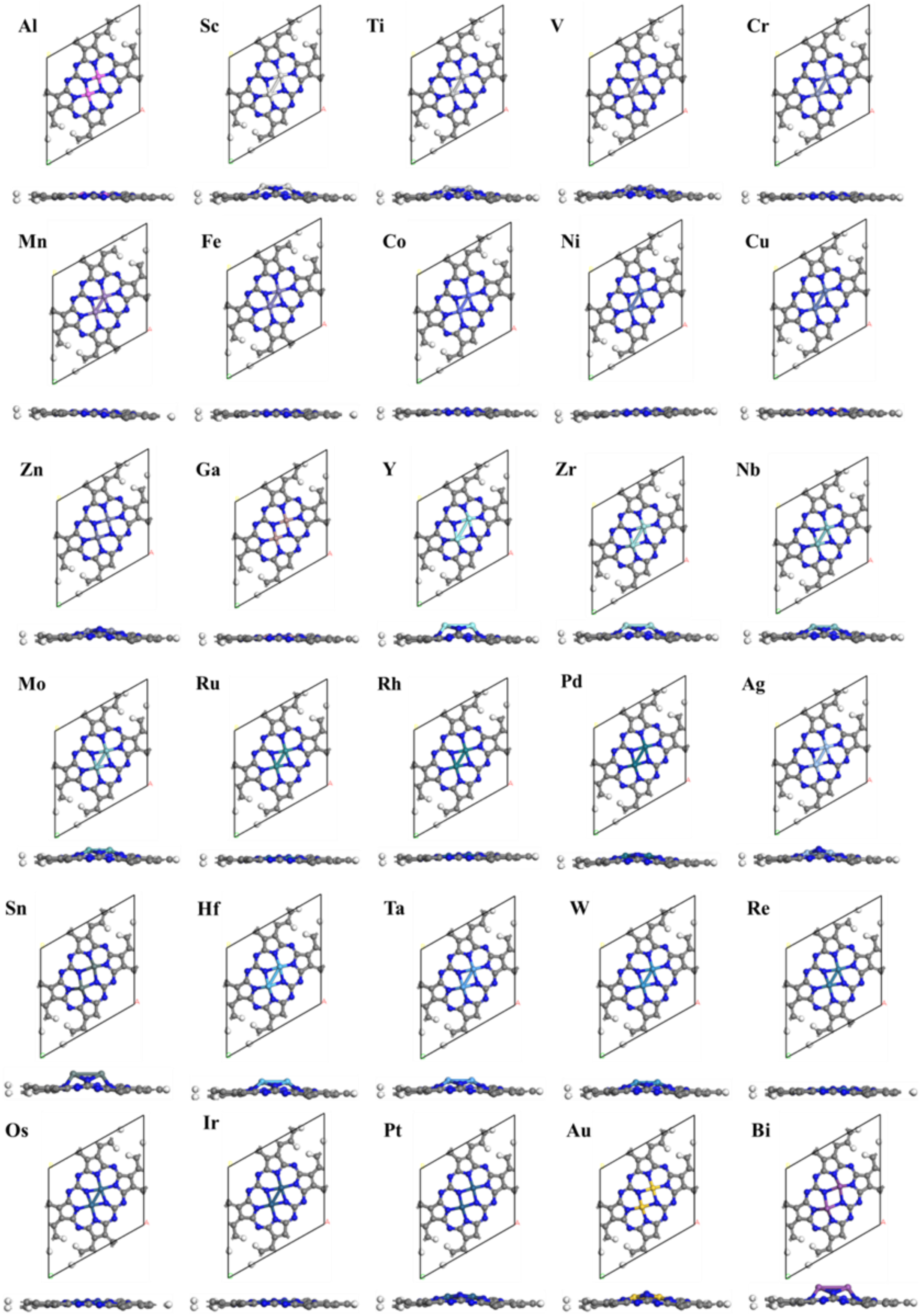

Figure S2. Top and side views of the optimized M_2-Pc monolayers.

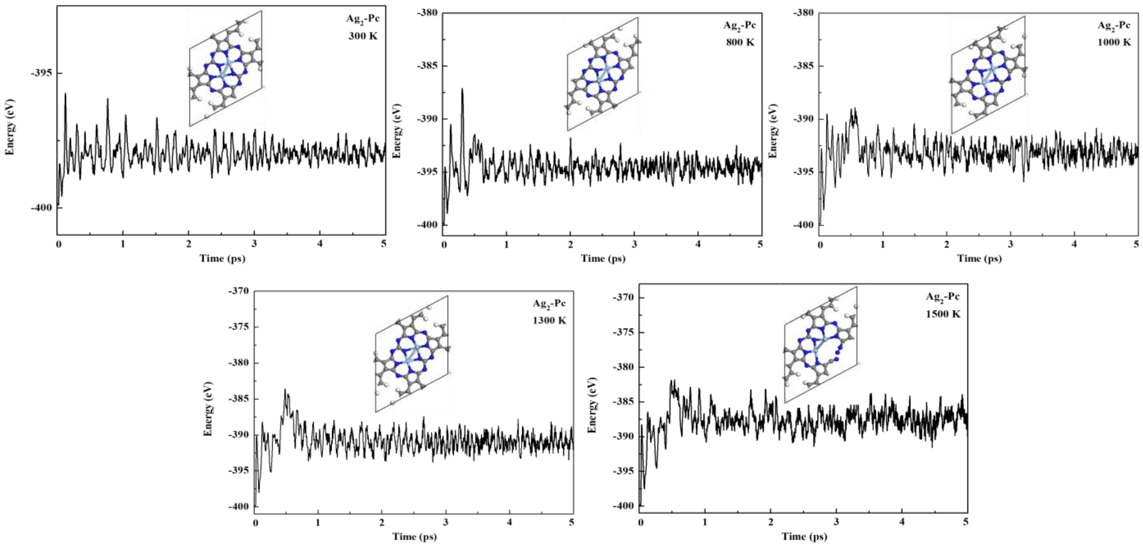

Figure S3. The energy evolution with time progress of the 5 ps FPMD simulation of the Ag_2-Pc at 300 K, 800, 1000, 1300, and 1500 K, as well as the snapshot of structure at the end of 5 ps.

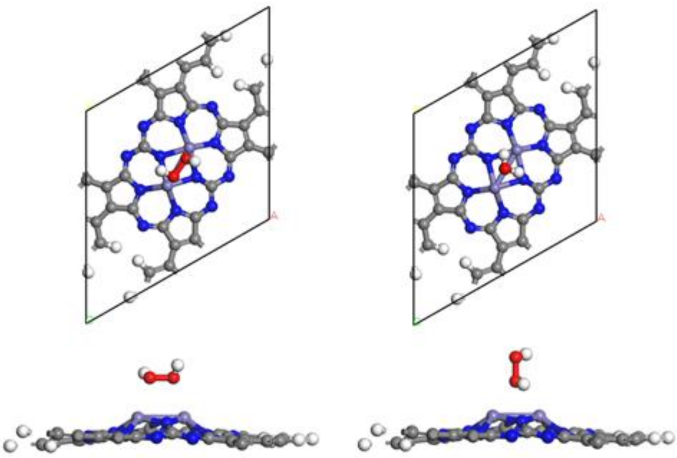

Figure S4. Two adsorption configurations of H_2O_2 on the M_2-Pc.

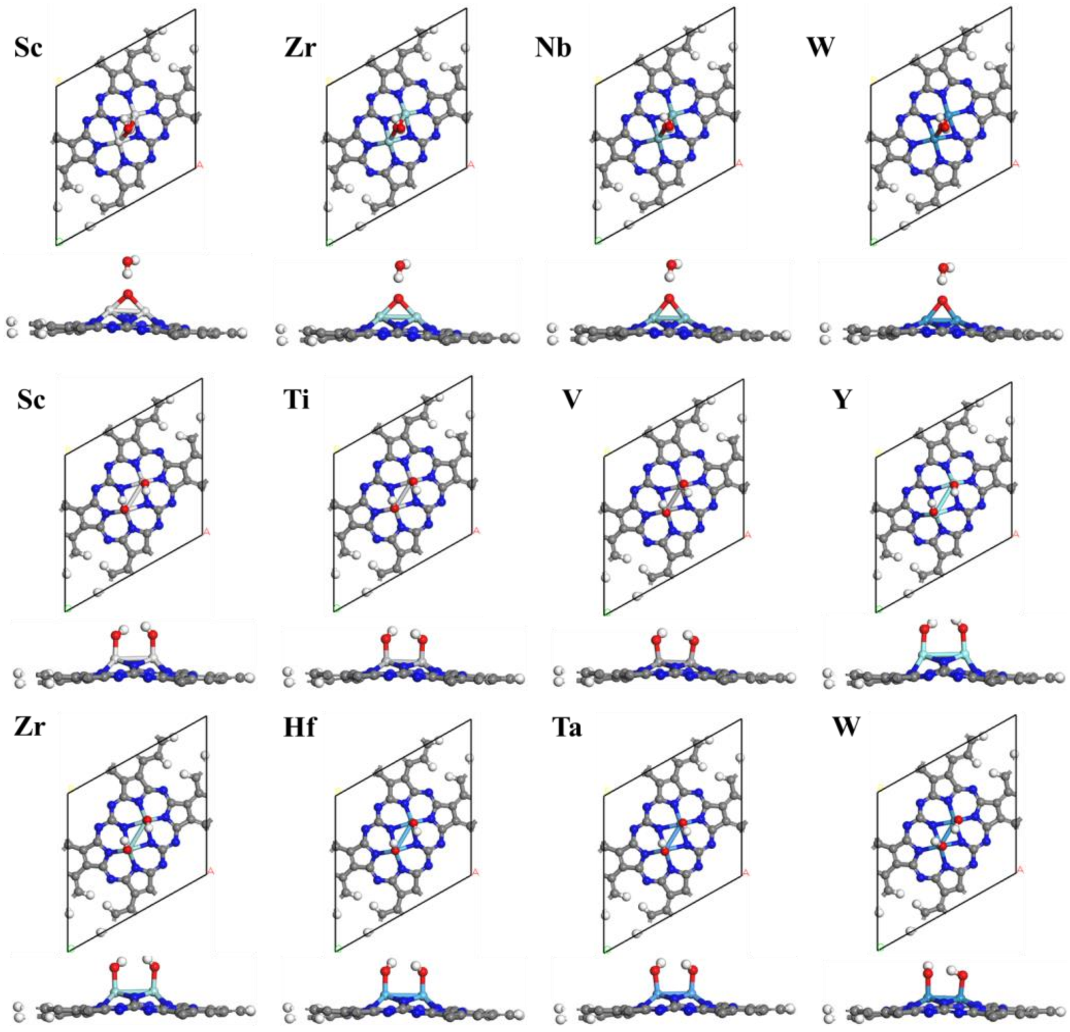

Figure S5. The structures of spontaneously dissociated H_2O_2 on the M_2-Pc monolayers.

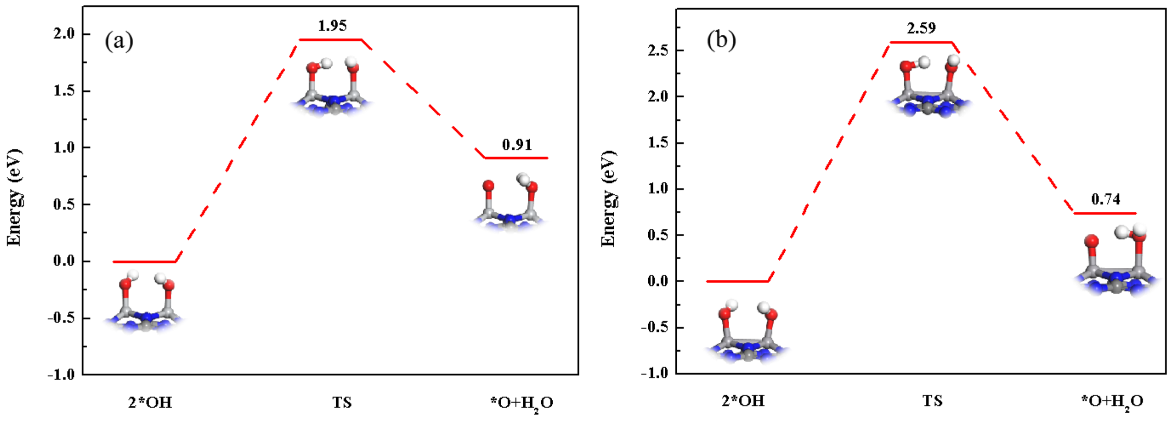

Figure S6. Transformation process of $*OH + *OH \rightarrow *O + H_2O$ on the Ti_2-Pc (**a**) and V_2-Pc (**b**) surfaces. The inset was the atomic structure model of each step.

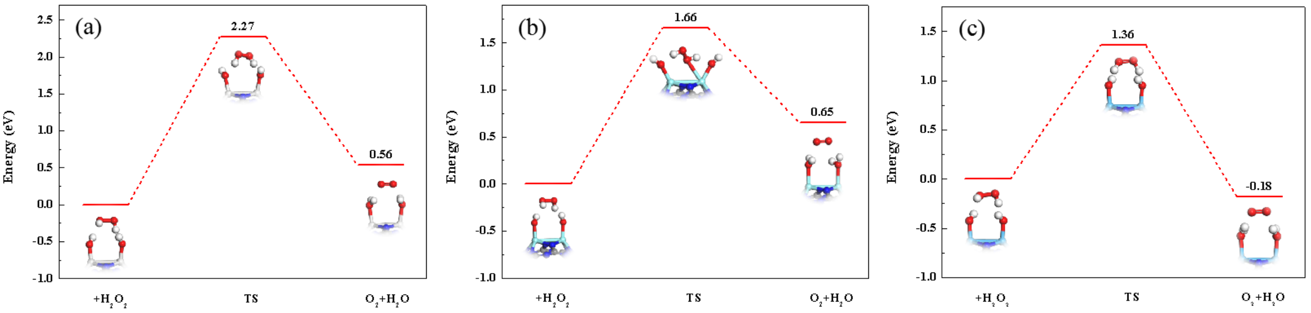

Figure S7. Transformation process of $H_2O_2 + 2(*OH) \rightarrow O_2 + 2H_2O$ on the Sc2-Pc (**a**), Y2-Pc (**b**), and Hf2-Pc surface (**c**). The inset was the atomic structure model of each step.

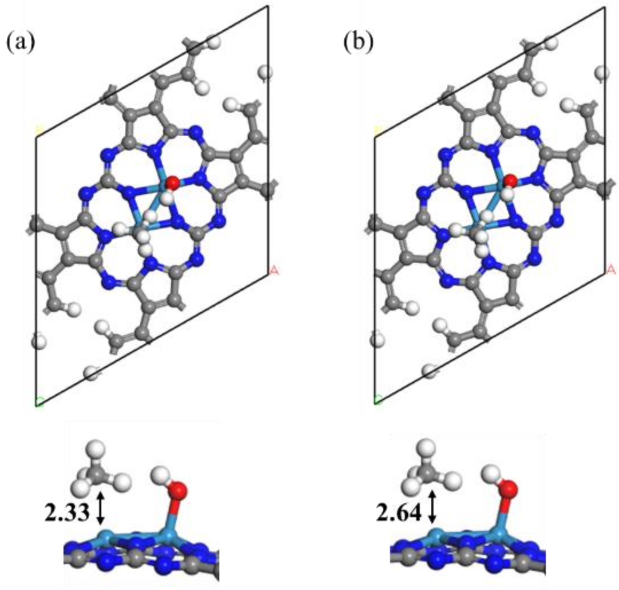

Figure S8. The initial state structure (**a**) and the final state structure (**b**) of methane adsorption on W2-Pc surface.

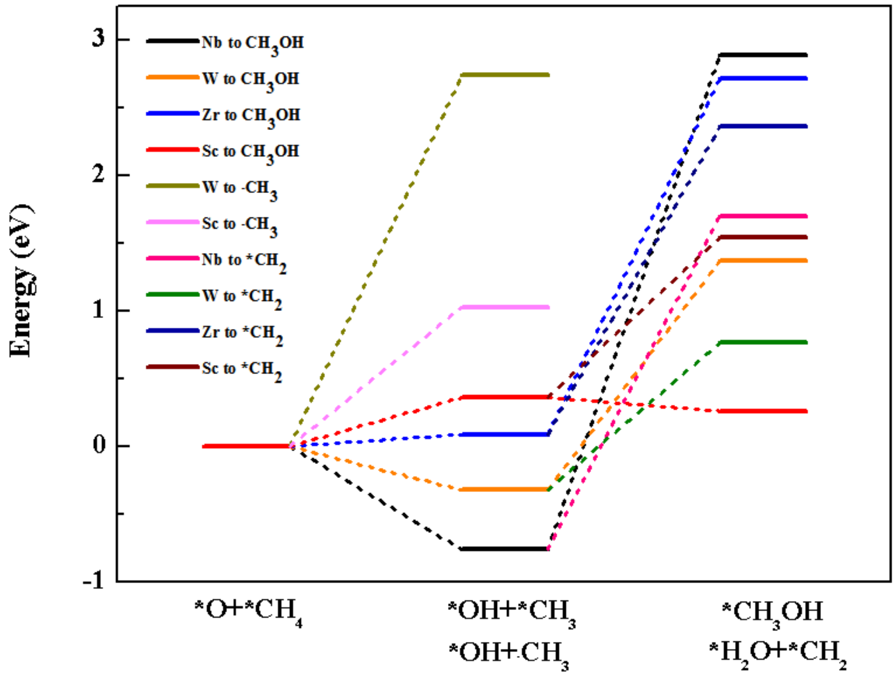

Figure S9. The corresponding energy profile of methane conversion *via* *O-assisted mechanism.

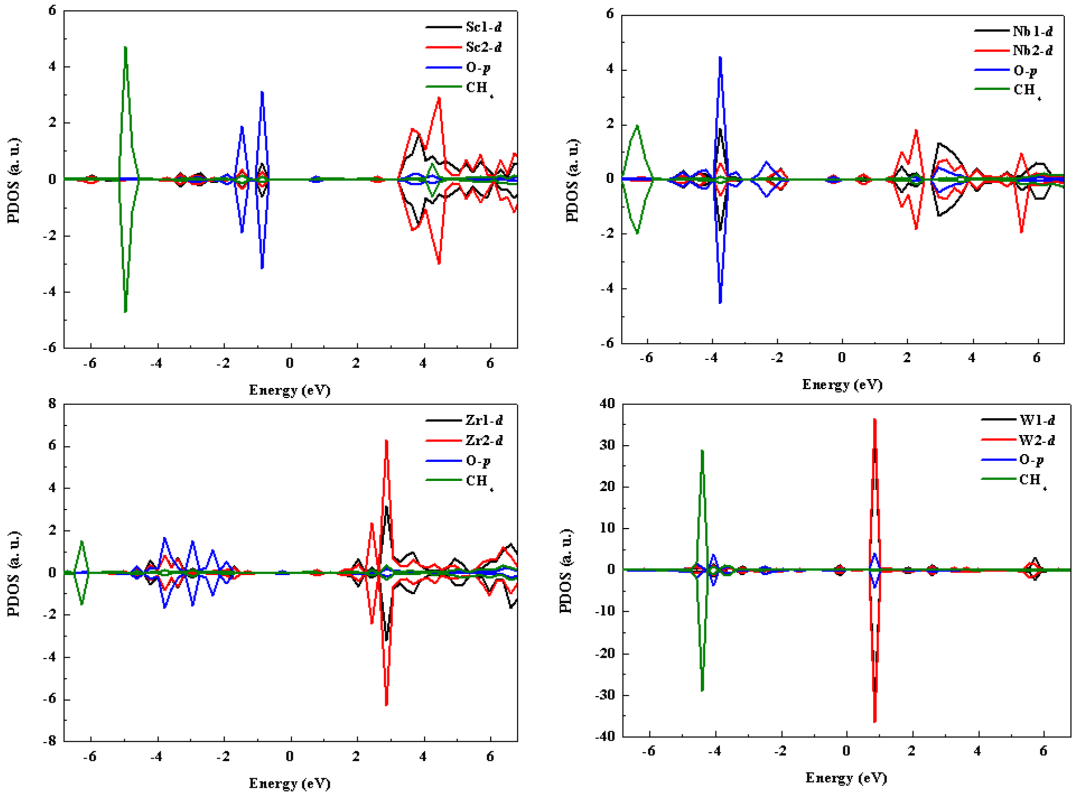

Figure S10. Partial density of states (PDOS) of CH_4 adsorption on M-O-M moiety. The Fermi level was set to zero.

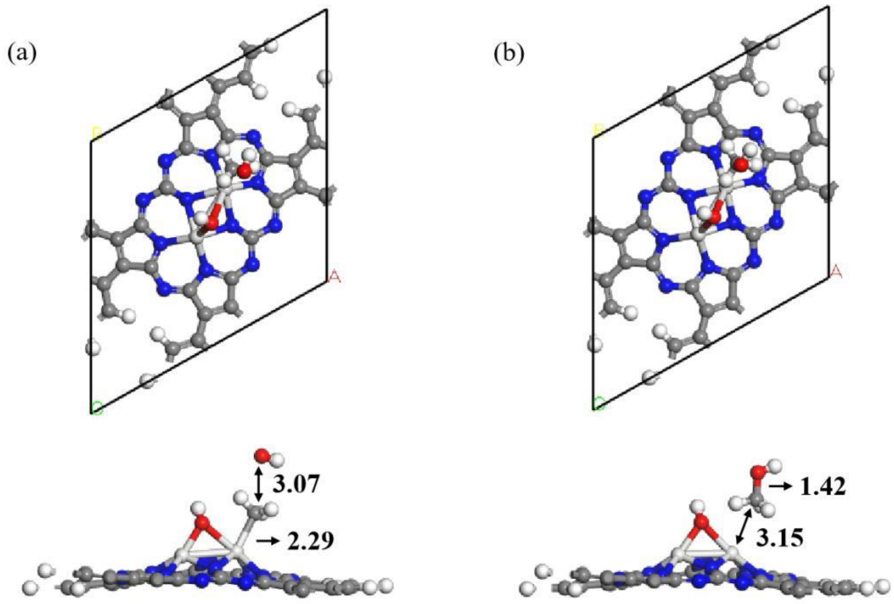

Figure S11. The initial state structure (**a**) and the final state structure (**b**) of the reaction between $*CH_3$ and OH in solution on Sc_2-Pc.

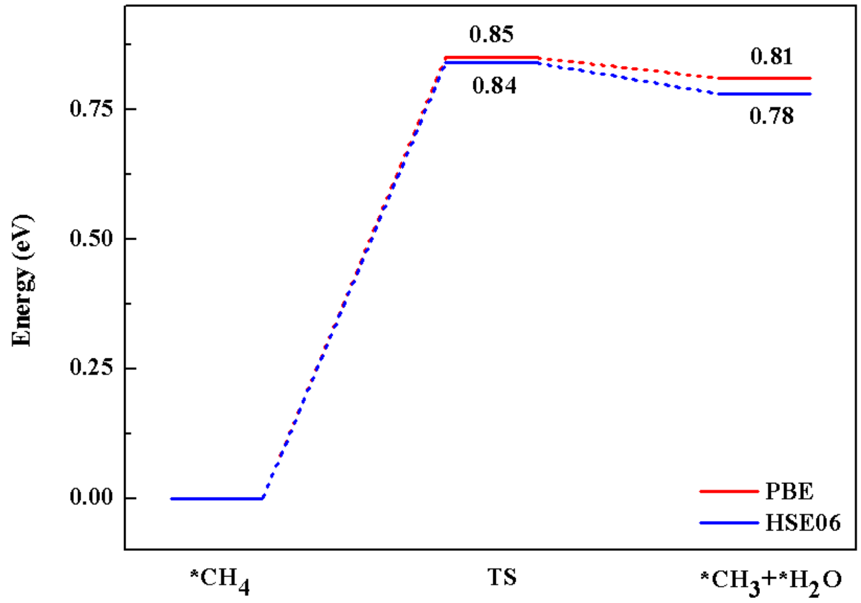

Figure S12. Energy diagram of the C−H bond cleavage on the Ti2-Pc surface calculated by PEB (red lines) and HSE06 functional (blue lines).
